# Patient Out-of-Pocket Costs for Type 2 Diabetes Medications When Aging Into Medicare

**DOI:** 10.1001/jamanetworkopen.2024.20724

**Published:** 2024-07-09

**Authors:** Douglas Barthold, Jing Li, Anirban Basu

**Affiliations:** 1 The Comparative Health Outcomes, Policy, and Economics Institute, School of Pharmacy, University of Washington, Seattle

## Abstract

**Importance:**

For people with type 2 diabetes (T2D), out-of-pocket medication costs may influence medication choice, adherence, and overall diabetes management and progression. Little is known about how these costs change as insured people enter Medicare at age 65 years, when coinsurance in the coverage gap and catastrophic phases of Part D coverage can be increased greatly by use of insulin and newer, branded medications (eg, dipeptidyl peptidase 4 inhibitors, glucagon-like peptide 1 agonists, and sodium-glucose cotransporter 2 inhibitors).

**Objective:**

To identify whether reaching age 65 years is associated with T2D medication out-of-pocket costs and utilization.

**Design, Setting, and Participants:**

This retrospective cohort study (2012-2020) featuring 7 years of follow-up used prescription drug claims data from the TriNetX Diamond Network. Participants included people in the US with diagnosed T2D, and claims for T2D medications were observed both before and after age 65 years. Data analysis was performed from October 2022 to September 2023.

**Exposure:**

Reaching age 65 years, according to participants’ year of birth.

**Main Outcomes and Measures:**

The primary outcome was patient out-of-pocket costs for T2D drugs per quarter (inflation adjusted to 2020 dollars). Utilization, measured as binary utilization of specific classes, and the number of claims for mutually exclusive classes and combinations of classes were also examined. All outcomes were examined using regression discontinuity design.

**Results:**

In claims data for 129 997 individuals with T2D diagnosed at ages 58 to 72 years (mean [SD] age, 65.50 [2.95] years; 801 235 female [50.9%]), reaching age 65 years was associated with an increase of $23.04 (95% CI, $19.86-$26.22) in mean quarterly out-of-pocket costs for T2D drugs, and an increase of $56.36 (95% CI, $51.48-$61.23) at the 95th percentile of spending, after utilization adjustment. Utilization decreased by 5.3% at age 65 years, from 3.40 claims per quarter (95% CI, 3.38-3.42 claims per quarter) to 3.22 claims per quarter (95% CI, 3.21-3.24 claims per quarter), but a shift in composition of utilization, including increased insulin use, was associated with additional increases in patient costs.

**Conclusions and Relevance:**

In this cohort study of individuals with T2D, the increase in spending upon reaching age 65 years (when most people enroll in Medicare) was associated with patient coinsurance in the coverage gap and catastrophic coverage phases of Medicare Part D. The increased patient cost burden at age 65 years and a modest reduction in overall T2D drug utilization suggest that as people with T2D age into Medicare, there is potentially an increase in nonadherence and diabetes complications.

## Key Points

**Question:**

What happens to out-of-pocket costs for type 2 diabetes (T2D) medications when people with T2D reach age 65 years (when most people enroll in Medicare), and what happens to their utilization of those medications?

**Findings:**

This cohort study with 129 997 individuals found that upon reaching age 65 years, quarterly out-of-pocket costs for T2D drugs increased by $23.04 at the mean and increased by $56.36 at the 95th percentile of spending, after adjustment for changes in utilization. Utilization decreased slightly at age 65 years (5.3% fewer claims per quarter), but a shift in composition of utilization, including increased insulin use, led to additional increases in patient costs.

**Meaning:**

These findings suggest that an increase in spending upon reaching age 65 years was associated with increased cost sharing in the coverage gap and catastrophic coverage phases of Medicare Part D, and likely resulted in worse adherence, worse T2D management, and increased T2D complications.

## Teaser

This cohort study examines the association of reaching age 65 years and enrolling in Medicare with out-of-pocket costs and utilization of type 2 diabetes medication.

## Introduction

More than 37 million people in the US have diabetes, representing 11.3% of the US population.^1^ It is the most expensive chronic condition in the US, with $237 billion in annual direct medical costs, and $90 billion in reduced productivity.^2^ Type 2 diabetes (T2D) makes up 90% to 95% of cases^3^ and is associated with dysfunction of the kidney, retina, cardiovascular system, neurons, and liver.^4,5^ Management strategies to alleviate the cost and health burdens include lifestyle modifications and prescription medicines; accordingly, the American Diabetes Association has published standards for pharmacologic approaches to glycemic control.^6^ However, patient out-of-pocket (OOP) costs for T2D medications pose a barrier to following these guidelines and can lead to nonadherence and ineffective management.^7,8^ In 2017, Medicare beneficiaries with diabetes spent an average of $928 OOP for their prescription drugs, and 40% to 60% of beneficiaries in the bottom income quartile had high OOP burden.^9^

Age is an important risk factor for diabetes, and 29% of Americans aged 65 years and older (16 million people) have diabetes.^1^ Medicare Part D coverage for these individuals includes cost sharing for antiglycemic medications,^10^ with a potentially large influence on medication choice, adherence, and T2D management and progression. These costs and the associated health outcomes have motivated policymakers to target OOP drug costs for Medicare beneficiaries with several provisions of the Inflation Reduction Act (IRA). For example, OOP costs for insulin were capped at $35 per month beginning in 2023. Other provisions target Part D OOP costs more generally, including a cap on total OOP costs at $3250 in 2024, and $2000 in 2025.^11^ These changes will effectively eliminate the Part D coverage gap, expand the initial coverage phase, and remove the 5% coinsurance rate in the catastrophic coverage phase. Coupled with provisions to reduce drug price growth and increased eligibility for the low-income subsidy for Part D, a substantial decrease in patient cost burden for medications is expected.^11,12^

Despite the importance of OOP costs for T2D prescription medicines and the justifiable interest of policymakers, little is known about how the cost burden changes for individuals as they reach age 65 years and enroll in Medicare. Commercial health insurance plans cover 70% of the insured population younger than 65 years in the US^13^; unlike Medicare Part D, effectively all these plans feature an OOP maximum and no coverage gap,^14^ which led us to hypothesize that transitioning to Medicare could increase OOP spending. This transition in coverage is a critical period, when medication choices and adherence can have long-term consequences for patients and the health care system. In this study, we quantify the increase in OOP costs for insured people with T2D when they reach age 65 years and most people enroll in Medicare. These trends have important implications for the expected effects of the IRA provisions: specifically, reduced cost sharing could improve T2D management and reduce complications.

## Methods

### Data and Setting

In this retrospective cohort study featuring an average of 7 years of follow-up, we examined 2012 to 2020 prescription drug claims data from the Diamond Network (TriNetX), restricted to individuals with a diagnosis of T2D (*International Classification of Diseases, Ninth Revision *code 250.xx or *International Statistical Classification of Diseases and Related Health Problems, Tenth Revision* code E11). The Diamond Network, described in other recent publications,^15,16^ features patient data from clearinghouses that capture 99% of US health plans and includes longitudinal diagnoses and prescribed medicines for more than 200 million patients. These aggregated data are sourced from commercial payers, Medicare, Medicaid, and the Veterans Administration.^17,18^ We examined claims at the quarterly level, and our analytic dataset featured patient-quarters with at least 1 claim with a National Drug Code (NDC) for a T2D drug. T2D generic drug names were linked to NDCs in the Food and Drug Administration NDC Directory product files.^19^ We restricted the sample to individuals who were observed both before and after age 65 years. Also, because we can only observe year of birth (not date of birth), we omitted the quarters from the calendar year when individuals reached age 65 years. In subgroup analyses, we used 3-digit zip code to link individuals to the Agency for Healthcare Research and Quality’s 2019 Social Determinants of Health Database.^20^ The details of the sample selection are in eTable 1 in Supplement 1. This study was not human participants research and therefore did not require institutional review board approval or informed consent, in accordance with the University of Washington institutional review board. This study complies with the Strengthening the Reporting of Observational Studies in Epidemiology (STROBE) reporting guidelines for cohort studies.^21^

### Outcomes and Measures

The primary outcome was patient OOP costs for T2D drugs per quarter (inflation adjusted to 2020 dollars). Age was approximated using year of birth, assuming all birthdays were July 1. We also examined utilization, measured as binary utilization of specific drug classes, as well as the number of claims for mutually exclusive classes and combinations of classes. T2D drugs included the following classes: biguanides (metformin), sulfonylureas, thiazolidinediones, insulin, dipeptidyl peptidase 4 (DPP4) inhibitors, glucagon-like peptide 1 (GLP1) receptor agonists, sodium-glucose cotransporter 2 (SGLT2) inhibitors, amylin analogs, α-glucosidase inhibitors, meglitinides, bile acid sequestrants, and dopamine-2 agonists. The full list of generic names for these classes is in the eAppendix in Supplement 1.

### Statistical Analysis

Data analysis was performed from October 2022 to September 2023. In unadjusted analyses, we calculated the mean and percentiles of OOP costs across age. In our primary analyses, we used generalized linear model regressions with a gamma distribution and log link to examine OOP costs and their association with reaching age 65 years. We also used quantile regressions to examine the associations of reaching age 65 years with the 95th percentile of OOP costs. All regressions featured a regression discontinuity (RD) design, with an indicator for being at least age 65 years as the variable of interest.^22^ The gamma and quantile regressions both adjusted for differential linear quarterly time trends before and after reaching age 65 years, year fixed effects, quarter fixed effects (accounting for seasonality and different phases of coverage), and utilization composition and intensity. Utilization composition and intensity were included as indicators for 1 to 3 claims and for at least 4 claims in each of these groups of drug classes: group 1 (metformin, sulfonylureas, thiazolidinediones, and insulin) monotherapies, group 1 duotherapies, group 2 (DPP4 inhibitors, GLP1 agonists, and SGLT2 inhibitors), and group 3 (all other classes and combinations). Group 1 monotherapies was the reference category. SEs were clustered at the individual level, and results are reported as average marginal effects (AMEs). In sensitivity analyses, we tested alternative sets of covariates, including a lack of utilization adjustment, adjustment for continuous claim counts for mutually exclusive classes, quadratic time trends, and class-specific linear time trends.

To examine heterogeneity in the association between reaching age 65 years and OOP costs for T2D drugs, we ran stratified analyses that divided the sample according to characteristics of the 3-digit zip codes where the individuals lived: above and below median income, above and below median percentage of residents enrolled in Medicaid, above and below the median percentage of Black residents, and above and below the median percentage of Hispanic residents. Although we are unable to observe insurance characteristics of individuals, these geographically stratified analyses provide insight on the degree to which results vary by race, ethnicity, insurance type, and income.

We analyzed the association between reaching age 65 years and utilization of T2D drugs using a Poisson regression with a dependent variable for number of T2D claims per quarter. We also used logistic regressions to examine binary dependent variables for any use of each specific class. Effects on utilization are reported as estimated margins below and above age 65 years. All utilization regressions featured an RD design, with an indicator for being at least age 65 years as the variable of interest and covariates for linear quarterly time trends before and after reaching age 65 years, year fixed effects, and quarter fixed effects. In a subsample analysis of utilization, we restricted the sample to individuals who ever had a quarter of OOP costs in the top 5% of OOP costs (≥$488.16). All regression analyses examined statistical significance using 2-sided *t *tests or *z *tests, with significance set at α = .05.

## Results

In a sample of 1 572 777 person-quarters, there were 129 997 unique individuals (mean [SD] age, 65.48 [2.95] years; 801 235 [50.9%] female) with a T2D diagnosis before the age of 65 years (median age, 66 years; range, 58-72 years) (eTable 1 in Supplement 1). Individuals were followed up for a mean of 29.3 quarters (range, 6-38 quarters), with a mean (SD) of 3.29 (2.71) T2D drug claims per quarter (Table 1). Unadjusted mean (SD) OOP costs for T2D drugs increased from $77.19 ($291.34) (median [IQR], $9.99 [$0.00-$45.24]) to $108.93 ($381.36) (median [IQR], $10.90 [$1.03-$61.80]) upon reaching age 65 years, and the increase was largely attributed to costs at the top end of the spending distribution (95th percentile increased from $337.44 to $508.20) (eTable 2 in Supplement 1). The Figure displays the path of mean spending and the 95th percentile of spending across year, stratified by birth cohort. OOP costs increased across age, regardless of birth cohort, and the costs continued to increase after age 65 years for every birth cohort. All individuals in the cohort had complete data for all measures in all quarters that were included in analyses (eTable 1 in Supplement 1). Our subsample for analyses stratified by 3-digit zip code omitted 21 649 individuals with missing zip code.

**Table 1.** Characteristics of Person-Quarters With a T2D Drug Claim, 2012-2020^a^

Characteristic	Participants, No. (%) (N = 1 572 777 patient-quarters)		
	All	Age <65 y	Age ≥65 y
Age, mean (SD), y	65.48 (2.95)	62.17 (1.44)	67.31 (1.72)
Sex			
Female	801 235 (50.9)	280 537 (50.2)	520 698 (51.4)
Male	771 542 (49.1)	278 539 (49.8)	493 003 (48.6)
Patient quarterly out-of-pocket sum, $			
All drugs			
Mean (SD)	220.13 (575.45)	196.02 (541.53)	233.42 (592.90)
Median (IQR)	74.20 (16.67-234.84)	76.32 (17.44-220.17)	72.90 (16.44-244.76)
T2D drugs			
Mean (SD)	101.10 (354.06)	76.31 (266.60)	114.77 (393.41)
Median (IQR)	10.60 (0.00-57.47)	11.30 (0.00-50.14)	9.54 (0.00-64.61)
Drug claims per quarter, mean (SD), No.			
All drugs	13.98 (11.17)	14.51 (11.27)	13.69 (11.10)
T2D drugs	3.29 (2.71)	3.47 (2.71)	3.18 (2.70)
T2D drug classes used per quarter, mean (SD), No.	1.56 (0.79)	1.56 (0.77)	1.57 (0.79)
Any claims for individual T2D drugs			
Metformin	1 103 410 (70.2)	402 665 (72.0)	700 745 (69.1)
Sulfonylureas	461 413 (29.3)	171 166 (30.6)	290 247 (28.6)
Thiazolidinediones	90 545 (5.8)	31 934 (5.7)	58 611 (5.8)
Insulin	397 613 (25.3)	137 241 (24.5)	260 372 (25.7)
Dipeptidyl peptidase 4	212 303 (13.5)	75 556 (13.5)	136 747 (13.5)
Glucagon-like peptide 1	91 500 (5.8)	24 253 (4.3)	67 247 (6.6)
Sodium-glucose cotransporter 2 inhibitor	76 454 (4.9)	16 662 (3.0)	59 792 (5.9)
Other T2D drugs	27 317 (1.7)	10 424 (1.9)	16 893 (1.7)

Abbreviation: T2D, type 2 diabetes.

^a^ Sample is TriNetX Diamond Network claims data for individuals with T2D (2012-2020), restricted to individuals who were observed both before and after age 65 years. Person-quarters in the calendar year when the individual reached age 65 years were omitted.

**Figure.**
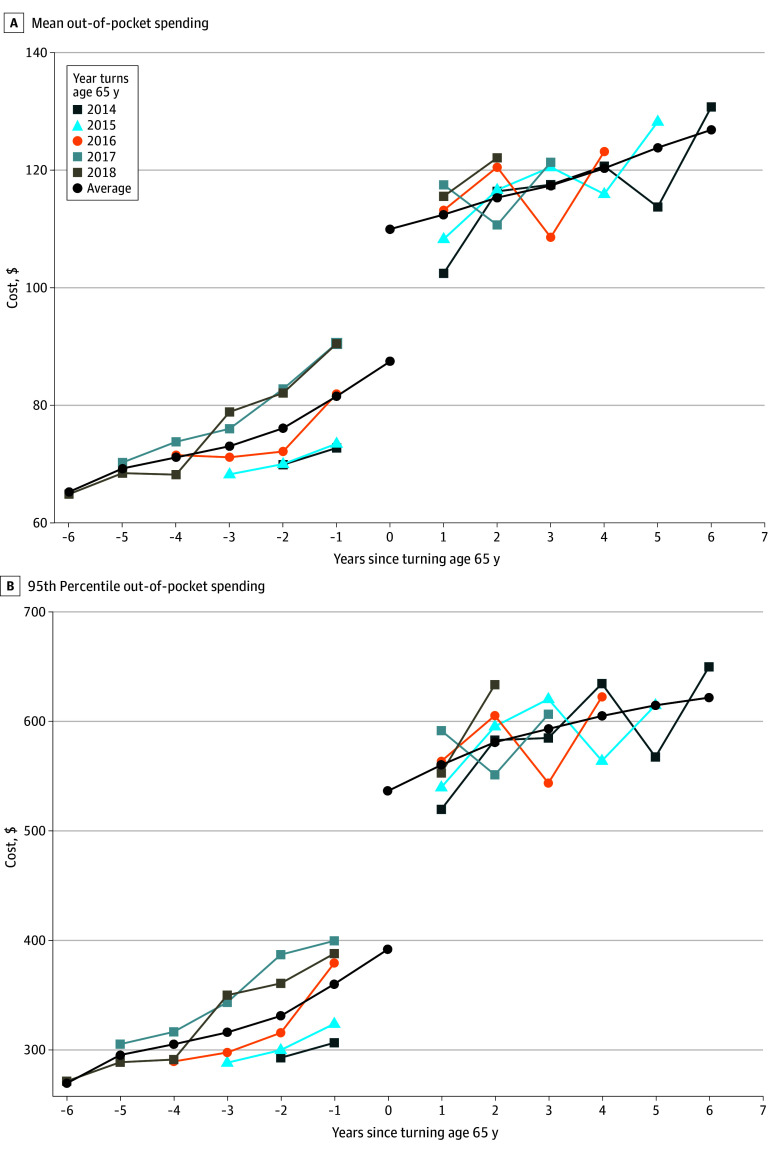
Quarterly Out-of-Pocket Spending on Type 2 Diabetes Drugs, Stratified by Birth Cohort The average trend is based on quadratic polynomial fit; values for the year participants reached age 65 years were extrapolated according to the relationships in the observed years, both before and after reaching age 65 years (N = 1 572 777 patient-quarters). The sample includes TriNetX Diamond Network claims data for individuals with type 2 diabetes (2012-2020), restricted to individuals who were observed both before and after reaching age 65 years. Person-quarters in the calendar year when the individual reached age 65 years were omitted.

### AME of Reaching Age 65 Years on OOP Spending

Our primary results show the AME of reaching age 65 years on quarterly OOP spending for type 2 diabetes drugs, adjusted for year, quarter, and compositional changes in drug utilization (Table 2). Reaching age 65 years was associated with an increase of approximately $23 in mean quarterly OOP costs for T2D drugs (AME, $23.04; 95% CI, $19.86-$26.22), demonstrating that enrolling in Medicare is associated with increased spending. The effect on the 95th percentile of OOP spending was larger, and was at least partially related to shifts in utilization. Without adjustment for utilization, the OOP spending increase at age 65 was approximately $134 (AME, $133.65; 95% CI, $124.28-$143.02), but with utilization adjustment, the increase was approximately $56 (AME, $56.36; 95% CI, $51.48-$61.23), suggesting that more than one-half of the increase at the 95th percentile can be attributed to utilization changes. The full regression results are in eTables 3 to 6 in Supplement 1. A series of sensitivity analyses confirmed the main findings, with the following alterations to the covariates: quadratic time trends (eTable 7 in Supplement 1), continuous claim count utilization adjustment (eTable 8 in Supplement 1), and class-specific time trends (eTable 9 in Supplement 1). Also, the use of insulin, DPP4 inhibitors, GLP1 agonists, and SGLT2 inhibitors is associated with much higher OOP costs (eTable 8 in Supplement 1).

**Table 2.** AME of Reaching Age 65 Years on Quarterly Out-of-Pocket Costs for Type 2 Diabetes Prescription Drugs^a^

Model	AME (95% CI)	*P* value
Adjusting for utilization^b^		
Mean OOP costs^c^	23.04 (19.86-26.22)	<.001
95th Percentile OOP costs^d^	56.36 (51.48-61.23)	<.001
Not adjusting for utilization		
Mean OOP costs	20.47 (17.26-23.67)	<.001
95th Percentile OOP costs	133.65 (124.28-143.02)	<.001

Abbreviations: AME, average marginal effect; OOP, out-of-pocket.

^a^ The sample is TriNetX Diamond Network claims data for individuals with type 2 diabetes (2012-2020), restricted to individuals who were observed both before and after age 65, and to quarters with at least 1 claim. Person-quarters in the calendar year when the individual turned 65 were omitted.

^b^ Utilization adjustment is indicators for 1 to 3 claims and 4 or more claims, in each of these groups of classes: group 1 (metformin, sulfonylureas, thiazolidinediones, and insulin) monotherapies, group 1 duotherapies, group 2 (dipeptidyl peptidase 4 inhibitors, glucagon-like peptide 1 agonists, sodium-glucose cotransporter 2 inhibitors), and group 3 (all other classes and combinations).

^c^ AMEs for mean OOP costs were estimated with generalized linear model regressions (gamma distribution, log-link) with a dependent variable for total quarterly out-of-pocket costs (2020 dollars) for type 2 diabetes medications, adjusted for linear quarterly trends before and after age 65 years, year fixed effects, and quarter fixed effects. SEs clustered at the individual level.

^d^ AMEs for 95th percentile costs were estimated with quantile regressions with a dependent variable for total quarterly out-of-pocket costs (2020 dollars) for type 2 diabetes medications, adjusted for linear quarterly trends before and after age 65 years, year fixed effects, and quarter fixed effects. SEs clustered at the individual level.

The association between reaching age 65 years and OOP costs was relatively consistent across 3-digit zip codes with varying income, racial, and ethnic characteristics (Table 3). The increase in spending was slightly higher in higher income zip codes and zip codes with a lower percentage of the population enrolled in Medicaid.

**Table 3.** AMEs of Reaching Age 65 Years on OOP Costs for Type 2 Diabetes Prescription Drugs, in Samples Stratified by 3-Digit Zip Code Characteristics^a^

Sample^b^	Participants, No.	AME (95% CI)	*P* value
Mean OOP spending^c^			
Overall	1 315 451	23.03 (19.55-26.51)	<.001
Income			
Above median	756 145	25.20 (20.48-29.92)	<.001
Below median	554 401	19.97 (14.85-25.10)	<.001
Percentage of population enrolled in Medicaid			
Above median	832 116	19.95 (15.57-24.34)	<.001
Below median	482 207	26.35 (20.92-31.79)	<.001
Percentage Black population			
Above median	708 562	23.25 (18.84-27.67)	<.001
Below median	604 886	22.97 (17.31-28.63)	<.001
Percentage Hispanic population			
Above median	653 101	21.91 (17.36-26.46)	<.001
Below median	660 545	24.57 (19.24-29.90)	<.001
95th Percentile OOP spending^d^			
Overall	1 315 451	55.86 (50.74-60.98)	<.001
Income			
Above median	756 145	58.86 (52.01-65.71)	<.001
Below median	554 401	51.09 (43.55-58.64)	<.001
Percentage of population enrolled in Medicaid			
Above median	832 116	50.94 (44.46-57.43)	<.001
Below median	482 207	56.72 (49.46-63.98)	<.001
Percentage Black population			
Above median	708 562	53.28 (47.22-59.35)	<.001
Below median	604 886	57.87 (49.14-66.61)	<.001
Percentage Hispanic population			
Above median	653 101	50.17 (43.82-56.52)	<.001
Below median	660 545	56.52 (48.47-64.56)	<.001

Abbreviations: AME, average marginal effect; OOP, out-of-pocket.

^a^ All regressions adjust for linear quarterly trends before and after age 65 years, year fixed effects, and quarter fixed effects. Utilization adjustment is indicators for 1 to 3 claims, and 4 or more claims, in each of these groups of drug classes: group 1 (metformin, sulfonylureas, thiazolidinediones, and insulin) monotherapies, group 1 duotherapies, group 2 (dipeptidyl peptidase 4 inhibitors, glucagon-like peptide 1 agonists, sodium-glucose cotransporter 2 inhibitors), and group 3 (all other classes and combinations). SEs clustered at the individual level.

^b^ Sample is TriNetX Diamond Network claims data for individuals with type 2 diabetes (2012-2020), restricted to individuals who were observed both before and after age 65 years, and to quarters with at least 1 claim. Person-quarters in the calendar year when the individual reached age 65 years were omitted.

^c^ AMEs for mean OOP costs were estimated with generalized linear model regressions (gamma distribution, log-link) with a dependent variable for total quarterly out-of-pocket costs (2020 dollars) for type 2 diabetes medications.

^d^ AMEs for 95th percentile costs were estimated with quantile regressions with a dependent variable for total quarterly out-of-pocket costs (2020 dollars) for type 2 diabetes medications.

### Association of Reaching Age 65 Years With Utilization

In addition to the shifts in unadjusted utilization shown in Table 1, the results of our adjusted analyses of T2D drug utilization are reported in Table 4. Upon reaching age 65 years, individuals reduced their number of claims per quarter by 5.3%, from 3.40 claims per quarter (95% CI, 3.38-3.42 claims per quarter) to 3.22 claims per quarter (95% CI, 3.21-3.24 claims per quarter). In a subsample of people with especially high spending, there was a 5.6% reduction in the number of claims, from 4.43 claims per quarter (95% CI, 4.38-4.48 claims per quarter) to 4.18 claims per quarter (95% CI, 4.15-4.22 claims per quarter). The characteristics of the high spending subsample are in eTable 10 in Supplement 1.

**Table 4.** Estimated Quarterly Utilization of T2D Drugs for Individuals Younger Than 65 Years and Age 65 Years and Older^a^

Utilization measure	Full sample^b^		Subsample of high spenders^c^	
	Age <65 y	Age ≥65 y	Age <65 y	Age ≥65 y
T2D drug claims, mean (95% CI), No.^d^	3.40 (3.38-3.42)	3.22 (3.21-3.24)	4.43 (4.38-4.48)	4.18 (4.15-4.22)
Drug class utilization, mean (95% CI), %^e^				
Metformin	70.6 (70.2-71.0)	69.9 (69.6-70.2)	60.0 (59.2-60.8)	59.9 (59.4-60.5)
Sulfonylureas	29.2 (28.8-29.6)	29.4 (29.1-29.7)	30.5 (29.8-31.3)	30.9 (30.3-31.5)
Insulin	24.7 (24.4-25.1)	25.6 (25.3-25.9)	40.6 (39.8-41.5)	43.0 (42.4-43.6)
Thiazolidinediones	5.6 (5.4-5.8)	5.9 (5.7-6.0)	6.8 (6.4-7.2)	7.1 (6.8-7.4)
Dipeptidyl peptidase 4 inhibitors	13.4 (13.0-13.7)	13.6 (13.4-13.8)	19.5 (18.8-20.2)	20.3 (19.8-20.8)
Glucagon-like peptide 1 agonists	5.9 (5.7-6.2)	5.8 (5.6-5.9)	11.6 (11.0-12.2)	11.5 (11.1-11.9)
Sodium-glucose cotransporter 2 inhibitors	5.7 (5.4-6.0)	5.1 (5.0-5.2)	11.4 (10.7-12.1)	9.7 (9.4-10.0)
Other T2D drugs	1.7 (1.6-1.8)	1.8 (1.7-1.8)	2.3 (2.1-2.6)	2.4 (2.2-2.6)

Abbreviation: T2D, type 2 diabetes.

^a^ Sample is TriNetX Diamond Network claims data for individuals with T2D (2012-2020), restricted to individuals who were observed both before and after age 65 years. Person-quarters in the calendar year when the individual reached age 65 years were omitted.

^b^ Full sample is 1 572 777 patient-quarters (except sodium-glucose cotransporter 2 inhibitors, which omits 2012 observations and has 1 467 728 person-quarters).

^c^ Subsample of high spenders is all patient-quarters for individuals who ever had out-of-pocket costs in the top 5% of out-of-pocket costs, and is 432 154 patient-quarters (except sodium-glucose cotransporter 2 inhibitors, which omits 2012 observations and has 401 703 person-quarters).

^d^ Number of T2D claims per quarter was estimated with a Poisson regression (estimated number of claims reported), adjusted for linear quarterly trends before and after age 65 years, year fixed effects, and quarter fixed effects. SEs clustered at the individual level.

^e^ Binary utilization of each class in each quarter was estimated in separate logistic regressions (estimated probability of use reported), adjusted for linear quarterly trends before and after age 65 years, year fixed effects, and quarter fixed effects. SEs clustered at the individual level.

Although the absolute changes in utilization were modest, they were large enough to affect OOP costs for enrollees with the highest spending, as evidenced by the OOP spending results shown in Table 2, which show that more than one-half of the increased spending observed upon reaching age 65 years can be attributed to utilization changes. This is likely attributable to the larger increase in insulin use at age 65 years for the highest spenders, from 40.6% of quarters (95% CI, 39.8%-41.5% of quarters) to 43.0% of quarters (95% CI, 42.4%-43.6% of quarters). Insulin was one of the most expensive classes, causing an increase of $131.03 per claim (eTable 8 in Supplement 1).

## Discussion

This cohort study examined OOP costs for diabetes medications for insured individuals with T2D, before and after they reached age 65 years. After adjusting for drug utilization and other confounders, we found that quarterly OOP costs increased by approximately $23 on average after age 65 years. The increase in costs at age 65 years was much larger at the top of the spending distribution, where the 95th percentile of unadjusted spending increased from approximately $337 to $508. Some of this increase can be attributed to changes in the composition of drug utilization, which shifted between classes at age 65 years and included an increase in insulin use; if we hold utilization and other characteristics constant, the adjusted increase in spending at the 95th percentile of spending was approximately $56. This shows that for the highest spenders it is both the transition to Medicare and compositional changes in utilization that are associated with the increase in spending at age 65 years.

Before age 65 years, the majority of insured people were enrolled in commercial plans that, unlike Medicare Part D, featured OOP maximums and no coverage gap^13,14^; it is these features that likely caused the observed spending increases at age 65 years, especially at the top end of the spending distribution. The increased patient cost burden at age 65 years and a modest reduction in overall T2D drug utilization suggest that as insured people with T2D reach age 65 years (and most enroll in Medicare), there is potentially an increase in nonadherence and diabetes complications.^7,8^ Increased OOP costs are especially concerning for individuals from racial and ethnic minoritized groups and low-income individuals, all of whom are more likely to experience nonadherence^23,24^ and have increased T2D complications and mortality.^25^ It is important to note that these increases in spending were found despite the closing of the Medicare Part D coverage gap during the study period.

The increase in OOP costs was slightly smaller among patients residing in zip codes with higher Medicaid enrollment and in zip codes with lower income. This is likely because cost sharing is lower for dual-eligible enrollees, who are partially insulated from the high costs of the coverage gap and catastrophic coverage phases of Medicare Part D.^26^ Despite this protection, the increases in this group were still substantial and constitute a considerable burden for low-income individuals. Although our data do not include individual-level insurance characteristics, the geographically stratified results, which show a discontinuity in OOP costs at age 65 years in all regions, give confidence that the transition to Medicare Part D at age 65 years led to the cost increase.

With the passage of the IRA, total OOP spending on all Part D drugs will be capped at $3250 in 2024 and $2000 in 2025.^11^ The OOP maximums of $3250 and $2000, if applied to our analytic sample in 2019 (78 163 individuals), would reduce spending for 3.4% and 9.0% of people (2689 and 7058), respectively. Coupled with the new $35 limit on insulin costs per month (which began in January 2023), we can expect the IRA to substantially reduce patient cost burden for people with T2D. Initial evidence on the effects of the $35 insulin cap in early 2023 show that it was associated with increased insulin fills.^27^ This aligns with earlier evidence, which has shown that reductions in insulin OOP costs are expected to lead to improved insulin adherence and large reductions in strokes, heart attacks, heart failure, and end-stage kidney disease.^28,29^ Another recent study^30^ estimated that for individuals with at least 1 cardiovascular risk factor, those spending more than $2000 per year would have a median reduction in OOP costs of $855 per year under the IRA.

Our results show that the use of insulin, DPP4 inhibitors, GLP1 agonists, and SGLT2 inhibitors are associated with much higher OOP costs. The IRA will reduce the cost sharing for these drugs, which will likely lead to increased utilization, and unless prices are reduced, it will also lead to higher cost to Medicare and taxpayers. Although the IRA’s Medicare drug price negotiation program has targeted 4 T2D drugs in its first year (insulin aspart, sitagliptin, empagliflozin, and dapagliflozin), the changes in price remain unknown.^31^

Relatedly, a previous study^32^ found that the insulin cost provision of the IRA is likely to reduce OOP costs for insulin by $500 per year for 1.6 million people, potentially leading to thousands of avoided complications and reduced mortality. The same study found that even after accounting for cost savings from avoided complications, the increased utilization of insulin at current prices ($7390 per year) is likely to lead to a $5.6 billion 20-year net increase in total medical spending. Substantial reductions in the price of insulin, which could occur via Medicare’s price negotiations, would be necessary to reach cost-effectiveness at a threshold of $100 000 per quality-adjusted life-year.^32^

### Strengths and Limitations

The strengths of this study include consistent observation of individuals both before and after they reach age 65 years, for an average of 7 years. Our large sample from the TriNetX Diamond Network is a good representation of insured people with T2D in the US. Importantly, the large sample allows a robust analysis of how costs changed at age 65 years, net of complex and diverse trajectories of utilization, as well as other confounders.

The study’s limitations include the possibility that we did not observe all claims of a given individual; TriNetX claims data have difficulty capturing individuals who leave the health care system and patients with inaccurate or changing diagnoses.^33^ Our results, however, which show an increase in OOP spending at age 65 years, are unlikely to be influenced by this limitation for 2 reasons. First, we only look at quarters with claims. Thus, for differential missingness across age 65 years to be associated with the observed cost increase after 65 years, there would need to be decreased missingness after age 65 years at the intensive margin within quarters, but our utilization results suggest the opposite of this: both adjusted and unadjusted claims per person per quarter decreased after age 65 years (Tables 1 and 3). Second, we found increased OOP spending only at the top end of the distribution (eTable 2 in Supplement 1). If there was decreased missingness upon reaching age 65 years, we would expect the median (and entire distribution) of OOP costs to increase as well, but this was not the case. Another limitation of our data are that we cannot observe insurance characteristics, and we must assume that individuals are transitioning to Medicare at age 65 years. This assumption, however, is valid for the vast majority of the population: according to the 2022 Medical Expenditure Panel Survey, 9.9% of the US population was enrolled in Medicare at age 64 years, compared with 90.9% at age 65 years.^34^ Another limitation of our data are that individual-level demographic details are limited. For example, the data include year of birth but not birthday; to address measurement error in age at the age 65 years cutoff, we drop all observations from the year that the individual turned 65 years. A limitation of the RD design in these data are that we must assume that there are no other changes occurring at age 65 years, aside from the transition to Medicare, that could influence our outcomes of interest. Additionally, our data lack clinical information regarding the severity of T2D for individuals in the sample, which is likely to influence medication utilization and OOP costs. However, it is biologically implausible that there would be a discontinuity in severity at age 65 years, meaning that the progression of T2D across time would not confound the results of RD analyses. Furthermore, there are limits on the generalizability of our findings: because we are examining claims for people before and after reaching age 65 years, we cannot make conclusions about people who were uninsured before age 65 years. Another study^35^ using the same data noted that academic medical centers were underrepresented, which also reduces generalizability.

## Conclusions

This study found that patient cost burden for T2D drugs is high and increases as insured people reach age 65 years and most enroll in Medicare. Our results have important implications for the provisions of the IRA, many of which aim to reduce these costs. Reduced patient cost burden will improve adherence and the management of T2D, likely leading to reductions in T2D complications. In addition, we expect these policies to increase utilization of expensive classes of drugs, some of which are targeted by upcoming Medicare price negotiations. Future research should clearly identify the value of new classes of T2D prescription drugs using data from clinical use, rather than trials, and should assess the impact of the IRA on OOP costs, drug adherence, and health outcomes.

## Notes

Supplement 1. **eTable 1. **Sample selection **eTable 2.** Quarterly out-of-pocket costs for type 2 diabetes medications by age **eTable 3.** Average marginal effects of patient and drug characteristics on mean out-of-pocket costs for type 2 diabetes prescription drugs, with utilization adjustment **eTable 4.** Average marginal effects of patient and drug characteristics on mean out-of-pocket costs for type 2 diabetes prescription drugs, without utilization adjustment **eTable 5.** Average marginal effects of patient and drug characteristics on the 95th percentile of out-of-pocket costs for type 2 diabetes prescription drugs, with utilization adjustment **eTable 6.** Average marginal effects of patient and drug characteristics on the 95th percentile of out-of-pocket costs for type 2 diabetes prescription drugs, without utilization adjustment **eTable 7.** Average marginal effects of patient and drug characteristics on mean out-of-pocket costs for type 2 diabetes prescription drugs, with linear and quadratic time trends **eTable 8.** Average marginal effects of patient and drug characteristics on mean out-of-pocket costs for type 2 diabetes prescription drugs, with continuous claim count utilization adjustment **eTable 9.** Average marginal effects of patient and medication characteristics on out-of-pocket costs for type 2 diabetes prescription drugs, including class-specific linear year trends **eTable 10.** Characteristics of subsample of individuals with OOP costs in the top 5% (No. (%)) **eAppendix.** List of type 2 diabetes prescription drug classes and molecule generic names

Supplement 2.
Data Sharing Statement
