# Supplementary material for: Patient Out-of-Pocket Costs for Type 2 Diabetes Medications When Aging Into Medicare
Source: JAMA Netw Open. 2024 Jul 9;7(7):e2420724. doi: 10.1001/jamanetworkopen.2024.20724 (PMC11234236; doi:10.1001/jamanetworkopen.2024.20724)
Supplement: Supplement 1. — eTable 1. Sample selection eTable 2. Quarterly out-of-pocket costs for type 2 diabetes medications by age eTable 3. Average marginal effects of patient and drug characteristics on mean out-of-pocket costs for type 2 diabetes prescription drugs, with utilization adjustment eTable 4. Average marginal effects of patient and drug characteristics on mean out-of-pocket costs for type 2 diabetes prescription drugs, without utilization adjustment eTable 5. Average marginal effects of patient and drug characteristics on the 95th percentile of out-of-pocket costs for type 2 diabetes prescription drugs, with utilization adjustment eTable 6. Average marginal effects of patient and drug characteristics on the 95th percentile of out-of-pocket costs for type 2 diabetes prescription drugs, without utilization adjustment eTable 7. Average marginal effects of patient and drug characteristics on mean out-of-pocket costs for type 2 diabetes prescription drugs, with linear and quadratic time trends eTable 8. Average marginal effects of patient and drug characteristics on mean out-of-pocket costs for type 2 diabetes prescription drugs, with continuous claim count utilization adjustment eTable 9. Average marginal effects of patient and medication characteristics on out-of-pocket costs for type 2 diabetes prescription drugs, including class-specific linear year trends eTable 10. Characteristics of subsample of individuals with OOP costs in the top 5% (No. (%)) eAppendix. List of type 2 diabetes prescription drug classes and molecule generic names [file jamanetwopen-e2420724-s001.pdf]

Supplemental Online Content

Barthold D, Li J, Basu A. Patient out-of-pocket costs for type 2 diabetes medications when aging into Medicare. *JAMA Netw Open*. 2024;7(7):e2420724. doi:10.1001/jamanetworkopen.2024.20724

eTable 1. Sample selection

eTable 2. Quarterly out-of-pocket costs for type 2 diabetes medications by age

eTable 3. Average marginal effects of patient and drug characteristics on mean out-of-pocket costs for type 2 diabetes prescription drugs, with utilization adjustment

eTable 4. Average marginal effects of patient and drug characteristics on mean out-of-pocket costs for type 2 diabetes prescription drugs, without utilization adjustment

eTable 5. Average marginal effects of patient and drug characteristics on the 95th percentile of out-of-pocket costs for type 2 diabetes prescription drugs, with utilization adjustment

eTable 6. Average marginal effects of patient and drug characteristics on the 95th percentile of out-of-pocket costs for type 2 diabetes prescription drugs, without utilization adjustment

eTable 7. Average marginal effects of patient and drug characteristics on mean out-of-pocket costs for type 2 diabetes prescription drugs, with linear and quadratic time trends

eTable 8. Average marginal effects of patient and drug characteristics on mean out-of-pocket costs for type 2 diabetes prescription drugs, with continuous claim count utilization adjustment

eTable 9. Average marginal effects of patient and medication characteristics on out-of-pocket costs for type 2 diabetes prescription drugs, including class-specific linear year trends

eTable 10. Characteristics of subsample of individuals with OOP costs in the top 5% (No. (%))

eAppendix. List of type 2 diabetes prescription drug classes and molecule generic names

This supplemental material has been provided by the authors to give readers additional information about their work.

© 2024 Barthold D et al. *JAMA Network Open.*

eTable 1. Sample selection

Sample	Unique individuals	Person-quarters
TriNetX individuals with type 2 diabetes (2012-2020)	2,136,019	34,818,802
Quarters with a claim for a T2D drug	1,147,436	11,764,976
Individuals observed both before and after age 65	132,591	1,831,602
Removing observations from the year they turned 65	129,998	1,572,976
Drop outliers	129,997	1,572,777
**Main analytic sample**	129,997	1,572,777
Sub-sample of high spending individuals	26,572	432,154
Sub-sample with non-missing 3-digit zip code	108,348	1,315,451

Individuals were identified as having diabetes if two or more International Classification of Diseases, Ninth Revision, Clinical Modification (ICD-9-CM) codes 250.xx or (ICD-10-CM) code E11 were present in any in-patient or out-patient records within a 12-month period. Outliers were quarters with patient payments over $100,000, charge over $500,000, or more than 30 claims for diabetes drugs. Sub-sample of high spending individuals are those who ever had a quarter with out-of-pocket costs in the top 5% of out-of-pocket costs (≥ $488.16).

eTable 2. Quarterly out-of-pocket costs for type 2 diabetes medications by age

Age	Mean	SD	5th %	25th %	50th %	75th %	95th %	N
All	101.10	354.06	0.00	0.00	10.60	57.47	488.16	1,572,777
60	72.04	223.03	0.00	3.27	13.56	54.50	303.46	49,699
61	73.75	246.83	0.00	1.15	13.08	52.32	306.29	83,604
62	76.22	259.19	0.00	0.00	11.30	49.95	333.00	123,621
63	79.98	287.76	0.00	0.00	11.10	49.95	343.92	170,868
64	77.19	291.34	0.00	0.00	9.99	45.24	337.44	101,534
65	108.93	381.36	0.00	1.03	10.90	61.80	508.20	134,235
66	110.07	415.24	0.00	0.00	9.54	59.40	548.28	264,824
67	113.58	364.77	0.00	0.00	9.09	61.80	574.61	210,956
68	117.29	390.94	0.00	0.00	8.83	64.89	589.92	160,479
69	121.46	394.10	0.00	0.00	8.95	73.44	608.96	114,270
70	123.35	416.50	0.00	0.00	8.95	77.25	625.60	73,696

Total quarterly patient spending (in 2020 dollars) for type 2 diabetes drugs, among people ages 58-73 with type 2 diabetes, in the TriNetX Diamond Network claims data (2012-2020), restricted to individuals who were observed both before and after age 65. Sample of person-quarters restricted to quarters where there was at least one claim for a type 2 diabetes drug. Person-quarters in the calendar year when the individual turned 65 were omitted. Values for age 64 were estimated using data from the two quarters immediately before the calendar year when individuals turned 65, and values for age 65 were estimated using data from the two quarters immediately after the calendar year when they turned 65, assuming July 1 birth date for everyone.

© 2024 Barthold D et al. *JAMA Network Open.*

eTable 3. Average marginal effects of patient and drug characteristics on mean out-of-pocket costs for type 2 diabetes prescription drugs, with utilization adjustment

Variable	AME	p-value	95% CI
Age ≥ 65	23.04	<0.001	(19.86-26.22)
Quarter trend < 65	0.43	0.002	(0.15-0.71)
Quarter trend ≥ 65	0.81	<0.001	(0.60-1.01)
Year fixed effects			
2012	0.00		
2013	-1.53	0.32	(-4.52-1.46)
2014	-9.85	<0.001	(-13.41--6.28)
2015	-8.02	<0.001	(-12.07--3.97)
2016	-6.65	0.004	(-11.16--2.13)
2017	-10.32	<0.001	(-15.14--5.51)
2018	-8.00	0.003	(-13.29--2.71)
2019	-18.20	<0.001	(-23.49--12.90)
2020	-11.65	<0.001	(-17.79--5.52)
Quarter fixed effects			
1st	0.00		
2nd	-7.60	<0.001	(-9.18--6.02)
3rd	-7.69	<0.001	(-9.36--6.02)
4th	-14.80	<0.001	(-16.57--13.03)
Utilization			
Group 1 monotherapy, 1-3 claims	0.00		
Group 1 monotherapy, ≥ 4 claims	182.48	<0.001	(176.41-188.55)
Group 1 duotherapy, 1-3 claims	13.38	<0.001	(12.40-14.35)
Group 1 duotherapy, ≥ 4 claims	99.67	<0.001	(96.02-103.31)
Group 2, 1-3 claims	99.17	<0.001	(96.88-101.46)
Group 2, ≥ 4 claims	279.43	<0.001	(271.53-287.32)
Group 3, 1-3 claims	89.92	<0.001	(87.32-92.52)
Group 3, ≥ 4 claims	251.42	<0.001	(246.81-256.02)

Results of a GLM regression (gamma distribution, log-link) with a dependent variable for total quarterly out-of-pocket costs (2020 dollars) for type 2 diabetes medications in quarters with at least 1 claim. N=1,572,777 patient-quarters. Sample is TriNetX Diamond Network claims data for individuals with type 2 diabetes (2012-2020), restricted to individuals who were observed both before and after age 65. Drug utilization variables are indicators for 1-3 claims and ≥ 4 claims in the noted class groups: group 1 (metformin, sulfonylureas, TZDs, and insulin), group 2 (DPP4 inhibitors, GLP1 agonists, SGLT2 inhibitors), group 3 (all other classes and combinations). Quarter trends are linear. Person-quarters in the calendar year when the individual turned 65 were omitted. Standard errors clustered at individual level. Abbreviations: AME (average marginal effect), GLM (generalized linear model), T2D (type 2 diabetes)

© 2024 Barthold D et al. *JAMA Network Open.*

eTable 4. Average marginal effects of patient and drug characteristics on mean out-of-pocket costs for type 2 diabetes prescription drugs, without utilization adjustment

Variable	AME	p-value	95% CI
Age ≥ 65	20.47	<0.001	(17.26-23.67)
Quarter trend < 65	0.26	0.09	(-0.04-0.56)
Quarter trend ≥ 65	0.52	<0.001	(0.32-0.72)
Year fixed effects			
2012	0.00		
2013	1.72	0.16	(-0.70-4.15)
2014	2.46	0.10	(-0.49-5.41)
2015	13.90	<0.001	(10.51-17.29)
2016	20.49	<0.001	(16.66-24.32)
2017	22.97	<0.001	(18.78-27.17)
2018	25.31	<0.001	(20.67-29.94)
2019	17.22	<0.001	(12.48-21.96)
2020	19.69	<0.001	(14.18-25.20)
Quarter fixed effects			
1st	0.00		
2nd	-6.67	<0.001	(-8.11--5.24)
3rd	-5.91	<0.001	(-7.50--4.32)
4th	-15.13	<0.001	(-16.77--13.5)

Results of a GLM regression (gamma distribution, log-link) with a dependent variable for total quarterly out-of-pocket costs (2020 dollars) for type 2 diabetes medications in quarters with at least 1 claim. N=1,572,777 patient-quarters. Sample is TriNetX Diamond Network claims data for individuals with type 2 diabetes (2012-2020), restricted to individuals who were observed both before and after age 65. Quarter trends are linear. Person-quarters in the calendar year when the individual turned 65 were omitted. Standard errors clustered at individual level. Abbreviations: AME (average marginal effect), GLM (generalized linear model), T2D (type 2 diabetes)

© 2024 Barthold D et al. *JAMA Network Open.*

eTable 5. Average marginal effects of patient and drug characteristics on the 95th percentile of out-of-pocket costs for type 2 diabetes prescription drugs, with utilization adjustment

Variable	AME	p-value	95% CI
Age ≥ 65	56.36	<0.001	(51.48-61.23)
Quarter trend < 65	0.54	<0.001	(0.25-0.82)
Quarter trend ≥ 65	2.83	<0.001	(2.48-3.18)
Year fixed effects			
2012	0.00		
2013	-5.48	0.01	(-9.50--1.46)
2014	-17.35	<0.001	(-21.86--12.84)
2015	-13.10	<0.001	(-18.11--8.09)
2016	-6.41	0.03	(-12.08--0.74)
2017	-6.98	0.03	(-13.16--0.81)
2018	5.94	0.12	(-1.57-13.45)
2019	-35.55	<0.001	(-42.18--28.93)
2020	13.92	0.01	(3.79-24.04)
Quarter fixed effects			
1st	0.00		
2nd	-13.41	<0.001	(-17.21--9.61)
3rd	-10.53	<0.001	(-14.31--6.75)
4th	-25.28	<0.001	(-28.87--21.68)
Utilization			
Group 1 monotherapy, 1-3 claims	0.00		
Group 1 monotherapy, ≥ 4 claims	936.73	<0.001	(916.00-957.45)
Group 1 duotherapy, 1-3 claims	53.34	<0.001	(50.05-56.63)
Group 1 duotherapy, ≥ 4 claims	447.57	<0.001	(437.39-457.75)
Group 2, 1-3 claims	371.85	<0.001	(366.31-377.39)
Group 2, ≥ 4 claims	1072.64	<0.001	(1047.72-1097.56)
Group 3, 1-3 claims	362.39	<0.001	(355.19-369.59)
Group 3, ≥ 4 claims	1016.11	<0.001	(1002.38-1029.83)

Results of a 95th percentile quantile regression with a dependent variable for total quarterly out-of-pocket costs (2020 dollars) for type 2 diabetes medications in quarters with at least 1 claim. N=1,572,777 patient-quarters. Sample is TriNetX Diamond Network claims data for individuals with type 2 diabetes (2012-2020), restricted to individuals who were observed both before and after age 65. Drug utilization variables are indicators for 1-3 claims and ≥ 4 claims in the noted class groups: group 1 (metformin, sulfonylureas, TZDs, and insulin), group 2 (DPP4 inhibitors, GLP1 agonists, SGLT2 inhibitors), group 3 (all other classes and combinations). Quarter trends are linear. Person-quarters in the calendar year when the individual turned 65 were omitted. Standard errors clustered at individual level. Abbreviations: AME (average marginal effect), T2D (type 2 diabetes)

© 2024 Barthold D et al. *JAMA Network Open.*

eTable 6. Average marginal effects of patient and drug characteristics on the 95th percentile of out-of-pocket costs for type 2 diabetes prescription drugs, without utilization adjustment

Variable	AME	p-value	95% CI
Age ≥ 65	133.65	<0.001	(124.28-143.02)
Quarter trend < 65	0.04	0.88	(-0.49-0.58)
Quarter trend ≥ 65	3.54	<0.001	(2.92-4.16)
Year fixed effects			
2012	0.00		
2013	6.43	0.09	(-0.97-13.83)
2014	23.03	<0.001	(15.09-30.97)
2015	85.51	<0.001	(76.19-94.84)
2016	109.65	<0.001	(99.32-119.97)
2017	127.51	<0.001	(115.96-139.06)
2018	153.93	<0.001	(140.35-167.50)
2019	87.71	<0.001	(75.25-100.17)
2020	133.65	<0.001	(115.82-151.48)
Quarter fixed effects			
1st	0.00		
2nd	-20.79	<0.001	(-27.79--13.79)
3rd	7.92	0.02	(1.09-14.74)
4th	-38.48	<0.001	(-44.98--31.97)

Results of a 95th percentile quantile regression with a dependent variable for total quarterly out-of-pocket costs (2020 dollars) for type 2 diabetes medications in quarters with at least 1 claim. N=1,572,777 patient-quarters. Sample is TriNetX Diamond Network claims data for individuals with type 2 diabetes (2012-2020), restricted to individuals who were observed both before and after age 65. Quarter trends are linear. Person-quarters in the calendar year when the individual turned 65 were omitted. Standard errors clustered at individual level. Abbreviations: AME (average marginal effect), T2D (type 2 diabetes)

© 2024 Barthold D et al. *JAMA Network Open.*

eTable 7. Average marginal effects of patient and drug characteristics on mean out-of-pocket costs for type 2 diabetes prescription drugs, with linear and quadratic time trends

Variable	AME	p-value	95% CI
Age ≥ 65	21.94	<0.001	(16.98-26.91)
Quarter trend < 65 (linear)	0.33	0.43	(-0.48-1.14)
Quarter trend ≥ 65 (linear)	0.00	0.78	(-0.04-0.03)
Quarter trend < 65 (quadratic)	1.15	<0.001	(0.65-1.64)
Quarter trend ≥ 65 (quadratic)	-0.01	0.15	(-0.03-0.00)
Year fixed effects			
2012	0.00		
2013	-1.55	0.32	(-4.58-1.49)
2014	-9.94	<0.001	(-13.55--6.34)
2015	-8.19	<0.001	(-12.28--4.11)
2016	-6.89	<0.001	(-11.45--2.33)
2017	-10.59	<0.001	(-15.44--5.75)
2018	-8.24	0.002	(-13.55--2.94)
2019	-18.28	<0.001	(-23.57--12.99)
2020	-11.71	<0.001	(-17.84--5.58)
Quarter fixed effects			
1st	0.00		
2nd	-7.66	<0.001	(-9.24--6.07)
3rd	-7.79	<0.001	(-9.45--6.13)
4th	-14.92	<0.001	(-16.70--13.15)
Utilization			
Group 1 monotherapy, 1-3 claims	0.00		
Group 1 monotherapy, ≥ 4 claims	182.50	<0.001	(176.43-188.57)
Group 1 duotherapy, 1-3 claims	13.38	<0.001	(12.40-14.35)
Group 1 duotherapy, ≥ 4 claims	99.66	<0.001	(96.02-103.31)
Group 2, 1-3 claims	99.16	<0.001	(96.87-101.45)
Group 2, ≥ 4 claims	279.44	<0.001	(271.55-287.34)
Group 3, 1-3 claims	89.91	<0.001	(87.31-92.51)
Group 3, ≥ 4 claims	251.41	<0.001	(246.81-256.02)

Results of a GLM regression (gamma distribution, log-link) with a dependent variable for total quarterly out-of-pocket costs (2020 dollars) for type 2 diabetes medications in quarters with at least 1 claim. N=1,572,777 patient-quarters. Sample is TriNetX Diamond Network claims data for individuals with type 2 diabetes (2012-2020), restricted to individuals who were observed both before and after age 65. Drug utilization variables are indicators for 1-3 claims and ≥ 4 claims in the noted class groups: group 1 (metformin, sulfonylureas, TZDs, and insulin), group 2 (DPP4 inhibitors, GLP1 agonists, SGLT2 inhibitors), group 3 (all other classes and combinations). Quarter trends are linear and quadratic. Person-quarters in the calendar year when the individual turned 65 were omitted. Standard errors clustered at individual level. Abbreviations: AME (average marginal effect), GLM (generalized linear model), T2D (type 2 diabetes)

© 2024 Barthold D et al. *JAMA Network Open.*

eTable 8. Average marginal effects of patient and drug characteristics on mean out-of-pocket costs for type 2 diabetes prescription drugs, with continuous claim count utilization adjustment

Variable	AME	p-value	95% CI
Age ≥ 65	18.03	<0.001	(10.72-25.35)
Quarter trend < 65	0.76	<0.001	(0.37-1.15)
Quarter trend ≥ 65	0.77	<0.001	(0.43-1.11)
Year fixed effects			
2012	0.00		
2013	-10.49	<0.001	(-14.77--6.20)
2014	-31.53	<0.001	(-36.45--26.61)
2015	-37.31	<0.001	(-43.10--31.52)
2016	-33.17	<0.001	(-40.64--25.71)
2017	-37.48	<0.001	(-47.49--27.47)
2018	-34.33	<0.001	(-43.80--24.86)
2019	-45.16	<0.001	(-53.74--36.58)
2020	-29.48	<0.001	(-39.84--19.13)
Quarter fixed effects			
1st	0.00		
2nd	-9.64	<0.001	(-13.02--6.27)
3rd	-10.31	<0.001	(-14.27--6.35)
4th	-20.28	<0.001	(-23.71--16.84)
Monotherapy claim counts			
Metformin	-1.65	0.22	(-4.28-0.99)
Sulfonylureas	6.73	<0.001	(4.78-8.69)
TZD	68.46	<0.001	(63.12-73.81)
Insulin	131.03	<0.001	(126.65-135.4)
DPP4 inhibitor	148.85	<0.001	(143.16-154.54)
GLP1 agonist	169.38	<0.001	(162.23-176.53)
SGLT2 inhibitors	150.24	<0.001	(144.07-156.41)
Duotherapy claim counts			
Metformin + Sulfonylureas	23.20	<0.001	(21.64-24.75)
Metformin + TZD	52.05	<0.001	(48.41-55.69)
Metformin + Insulin	92.52	<0.001	(89.63-95.40)
Sulfonylureas + TZD	44.05	<0.001	(40.48-47.63)
Sulfonylureas + Insulin	91.93	<0.001	(88.72-95.15)
TZD + Insulin	98.13	<0.001	(91.83-104.44)
Metformin + DPP4 inhibitor	107.82	<0.001	(104.24-111.39)
Metformin + SGLT2 inhibitor	109.52	<0.001	(105.75-113.29)
Metformin + GLP1 agonist	111.62	<0.001	(107.71-115.53)
Any other T2D claim count	89.41	<0.001	(86.82-91.99)

Results of a GLM regression (gamma distribution, log-link) with a dependent variable for total quarterly out-of-pocket costs (2020 dollars) for type 2 diabetes medications in quarters with at least 1 claim. N=1,572,777 patient-quarters. Sample is TriNetX Diamond Network claims data for individuals with type 2 diabetes (2012-2020), restricted to individuals who were observed both before and after age 65. Drug utilization variables are mutually exclusive claim counts, top-coded at 6 claims per quarter. Quarter trends are

© 2024 Barthold D et al. *JAMA Network Open.*

linear. Person-quarters in the calendar year when the individual turned 65 were omitted. Standard errors clustered at individual level. Abbreviations: AME (average marginal effect), GLM (generalized linear model), T2D (type 2 diabetes)

eTable 9. Average marginal effects of patient and medication characteristics on out-of-pocket costs for type 2 diabetes prescription drugs, including class-specific linear year trends

Variable	AME	p-value	95% CI
Age ≥ 65	12.03	<0.001	(7.05-17.02)
Quarter trend < 65	0.66	<0.001	(0.38-0.95)
Quarter trend ≥ 65	0.25	0.15	(-0.09-0.58)
Year fixed effects			
2012	0.00		
2013	-135.96	<0.001	(-152.01—119.91)
2014	-246.06	<0.001	(-268.96—223.16)
2015	-303.67	<0.001	(-330.51—276.82)
2016	-335.63	<0.001	(-365.57—305.70)
2017	-368.78	<0.001	(-401.82—335.74)
2018	-390.51	<0.001	(-424.76—356.27)
2019	-409.82	<0.001	(-443.16—376.49)
2020	-423.57	<0.001	(-458.89—388.26)
Quarter fixed effects			
1st	0.00		
2nd	-10.80	<0.001	(-14.26—7.35)
3rd	-11.39	<0.001	(-14.51—8.26)
4th	-17.66	<0.001	(-21.28—14.03)
Utilization			
Group 1 monotherapy, 1-3 claims	0.00		
Group 1 monotherapy, ≥ 4 claims	103.19	<0.001	(96.74-109.64)
Group 1 duotherapy, 1-3 claims	21.29	<0.001	(19.12-23.47)
Group 1 duotherapy, ≥ 4 claims	87.71	<0.001	(83.41-92.01)
Group 2, 1-3 claims	109.29	<0.001	(103.74-114.85)
Group 2, ≥ 4 claims	310.98	<0.001	(296.04-325.92)
Group 3, 1-3 claims	88.93	<0.001	(84.41-93.45)
Group 3, ≥ 4 claims	258.08	<0.001	(247.98-268.17)
Year*class interactions			
Monotherapies			
Metformin	0		
Sulfonylureas	5.33	<0.001	(4.39-6.28)
TZD	20.41	<0.001	(18.55-22.27)
Insulin	61.22	<0.001	(59.85-62.59)
DPP4 inhibitor	28.85	<0.001	(27.50-30.19)
GLP1 agonist	35.38	<0.001	(33.96-36.80)
SGLT2 inhibitors	33.49	<0.001	(32.05-34.93)

© 2024 Barthold D et al. *JAMA Network Open.*

Variable	AME	p-value	95% CI
Duotherapies			
Metformin + Sulfonylureas	3.27	<0.001	(1.85-4.70)
Metformin + TZD	14.45	<0.001	(12.07-16.83)
Metformin + Insulin	49.80	<0.001	(48.32-51.27)
Sulfonylureas + TZD	11.98	<0.001	(9.86-14.09)
Sulfonylureas + Insulin	48.06	<0.001	(46.38-49.74)
TZD + Insulin	52.25	<0.001	(46.94-57.57)
Metformin + DPP4 inhibitor	27.34	<0.001	(26.02-28.65)
Metformin + SGLT2 inhibitor	32.26	<0.001	(30.79-33.74)
Metformin + GLP1 agonist	33.28	<0.001	(31.76-34.80)
Any other T2D drug	32.59	<0.001	(31.39-33.78)

Results of a GLM regression (gamma distribution, log-link) with a dependent variable for total quarterly out-of-pocket costs (2020 dollars) for type 2 diabetes medications in quarters with at least 1 claim. N=1,572,777 patient-quarters. Sample is TriNetX Diamond Network claims data for individuals with type 2 diabetes (2012-2020), restricted to individuals who were observed both before and after age 65. Drug utilization variables are indicators for 1-3 claims and ≥ 4 claims in the noted class groups: group 1 (metformin, sulfonylureas, TZDs, and insulin), group 2 (DPP4 inhibitors, GLP1 agonists, SGLT2 inhibitors), group 3 (all other classes and combinations). Additionally, this regression adjusts for mutually exclusive class indicators interacted with year. Quarter trends are linear. Person-quarters in the calendar year when the individual turned 65 were omitted. Standard errors clustered at individual level. Abbreviations: AME (average marginal effect), GLM (generalized linear model), T2D (type 2 diabetes)

eTable 10. Characteristics of subsample of individuals with OOP costs in the top 5% (No. (%))

Characteristic	All	Age <65	Age ≥65
Age (mean (SD), years)	65.5 (3.01)	62.1 (1.45)	67.4 (1.75)
Female	200,845 (46.5)	69,382 (45.6)	131,463 (47.0)
Male	231,309 (53.5)	82,804 (54.4)	148,505 (53.0)
Patient quarterly OOP sum (all drugs) (mean (SD))	473.3 (884.21)	382.9 (808.83)	522.5 (918.88)
Patient quarterly OOP sum (T2D drugs) (mean (SD))	296.8 (627.99)	205.8 (479.26)	346.2 (690.61)
Total drug claims per quarter (mean (SD))	14.4 (10.43)	14.7 (10.60)	14.2 (10.33)
T2D drug claims per quarter (mean (SD))	4.3 (3.29)	4.4 (3.28)	4.2 (3.29)
T2D drug classes used per quarter (mean (SD))	1.8 (0.89)	1.8 (0.87)	1.9 (0.90)
Metformin - any claims	259,111 (60.0)	96,986 (63.7)	162,125 (57.9)
Sulfonylureas - any claims	132,964 (30.8)	50,340 (33.1)	82,624 (29.5)
TZD - any claims	30,238 (7.0)	10,400 (6.8)	19,838 (7.1)
Insulin - any claims	182,318 (42.2)	58,459 (38.4)	123,859 (44.2)
DPP4 - any claims	86,398 (20.0)	30,535 (20.1)	55,863 (20.0)
GLP1 - any claims	49,769 (11.5)	13,195 (8.7)	36,574 (13.1)
SGLT2 - any claims	40,259 (9.3)	9,275 (6.1)	30,984 (11.1)
Other T2D drugs - any claims	10,218 (2.4)	3,791 (2.5)	6,427 (2.3)

The subsample presented here is the main sample, restricted to patient-quarters for individuals who ever had OOP costs in the top 5% of OOP costs (≥$488.16), and is 432,154 patient-quarters (except SGLT2 inhibitors, which omits 2012 observations and has 401,703 person-quarters). Main sample is TriNetX Diamond Network claims data for individuals with type 2 diabetes (2012-2020), restricted to individuals who were observed both before and after age 65. Person-quarters in the calendar year when the individual turned 65 were omitted. Abbreviations: OOP (out-of-pocket), T2D (type 2 diabetes), SD (standard deviation)

eAppendix. List of type 2 diabetes prescription drug classes and molecule generic names

- Biguanides (metformin)
  - Molecules: metformin
- Insulin
  - Molecules: insulin
- Sulfonylureas
  - Molecules: chlorpropamide, glimepiride, glipizide, glyburide, tolazamide, tolbutamide
- Thiazolidinediones (TZDs)
  - Molecules: pioglitazone, rosiglitazone
- Dipeptidyl peptidase 4 (DPP4) inhibitors
  - Molecules: alogliptin, linagliptin, saxagliptin, sitagliptin
- Glucagon-like peptide 1 (GLP1) receptor agonists
  - Molecules: albiglutide, dulaglutide, exenatide, liraglutide, semaglutide, lixisenatide
- Sodium-glucose cotransporter-2 (SGLT2) inhibitors
  - Molecules: ertugliflozin, canagliflozin, dapagliflozin, empagliflozin
- Amylin analogs
  - Molecules: pramlintide
- Alpha-glucosidase inhibitors
  - Molecules: acarbose, miglitol
- Meglitinides
  - Molecules: nateglinide, repaglinide
- Bile acid sequestrants
  - Molecules: colesevelam
- Dopamine-2 agonists
  - Molecules: bromocriptine
