# Supplementary material for: Patient Out-of-Pocket Costs for Type 2 Diabetes Medications When Aging Into Medicare
Source: JAMA Netw Open. 2024 Jul 9;7(7):e2420724. doi: 10.1001/jamanetworkopen.2024.20724 (PMC11234236; doi:10.1001/jamanetworkopen.2024.20724)
Supplement: Supplement 2. — Data Sharing Statement [file jamanetwopen-e2420724-s002.pdf]

Data Sharing Statement

Barthold. Patient Out-of-Pocket Costs for Type 2 Diabetes Medications When Aging Into Medicare. *JAMA Netw Open*. Published July 09, 2024.
doi:10.1001/jamanetworkopen.2024.20724

Data
Data available: No
